# Iodine nutrition and papillary thyroid cancer

**DOI:** 10.3389/fnut.2022.1022650

**Published:** 2022-10-20

**Authors:** Xueqi Zhang, Fan Zhang, Qiuxian Li, Chuyao Feng, Weiping Teng

**Affiliations:** National Health Commission Key Laboratory of Diagnosis and Treatment of Thyroid Diseases, Department of Endocrinology and Metabolism, Institute of Endocrinology, The First Hospital of China Medical University, Shenyang, China

**Keywords:** iodine, papillary thyroid cancer, iodine nutrition, thyroid cancer, epidemiological studies

## Abstract

Thyroid cancer (TC) is the most frequent endocrine malignancy. The incidence of TC, especially papillary thyroid carcinoma (PTC), has continued to rise all over the world during the past few years, for reasons that are not entirely clear. Though the phenomenon of overdiagnosis is occurring, it is not the sole driver of the substantial increase in incidence. Lifestyle, environmental factors, or complications are considered to be potential risk factors. Among these factors, iodine is a micronutrient that is vital to thyroid function. The effect of iodine intake on PTC has been controversial for many years and the epidemiological or experimental studies provided diametrically opposite conclusions. Combining all these studies, we found that iodine nutrition may affect the overall prevalence, distribution of the histological types, and clinicopathological aggressiveness of TC, especially PTC. However, the available evidence is poor due to the impact of various internal and external related factors. Therefore, this article sums up available results from both epidemiological and experimental studies, future studies are also warranted to expound on the relationship between overall PTC prevalence and iodine intake.

## Introduction

Though the global thyroid cancer (TC) incidence has grown remarkably over the past few years (1–3), the mortality rate remains static (4, 5). There are four main kinds of TC: papillary thyroid carcinoma (PTC), follicular thyroid carcinoma (FTC), medullary thyroid carcinoma (MTC), and anaplastic thyroid cancer (ATC) (6). ATC, one of the fatal and rare forms of TC (1–2%) that generally presents as a rapidly growing neck tumor (7), needs early, accurate identification and timely treatment (8). However, most TC especially PTC differentiates well and has a low risk of becoming malignant. Thus, it is necessary to adjust the treatment plan according to the specific situation to avoid overtreatments and identify controllable risk factors to conduct preventive programs. Risk factors including radiation exposure, dietary nutrition, BMI (9), metabolic syndrome (10), environmental pollutants, family history of thyroid nodules, and overdiagnosis have been reported (11). Despite overdiagnosis, environmental/lifestyle factors do contribute to some increase in TC prevalence (12–16).

Dietary iodine intake has also been speculated as a risk factor that may influence the occurrence and development of PTC (17), but inconsistencies in research results have led to great controversy throughout these years. Iodine is a crucial micronutrient and a vital composition for the biosynthesis of thyroid hormone which plays a part in various biochemical and metabolic pathways throughout the human body (18, 19). The thyroid can maintain normal function, and keep thyroid hormone and thyroid stimulating hormone (TSH) in an appropriate ratio through automatic regulation even though daily iodine intake fluctuates widely. A U curve has been come up by many studies (20–22), both chronic iodine deficiency and excess can lead to thyroid dysfunction by interfering with homeostasis (23, 24), which means that the dose-based effects of iodine nutrition on the prevalence of TC need to be considered.

At the population level, the main sources of iodine intake include salt (25), water, milk, and seaweed (26, 27). The thyroid gland actively uptakes about 120 μg of iodine per day, which is distributed to a reservoir in the thyroid that contains about 5,000–10,000 μg of iodine. Monoiodothyronine and diiodothyronine are deiodinated in the periphery and T4 is converted to T3, resulting in the return of 60 μg of iodine per day to the external thyroid reservoir. Approximately 110 μg of iodine (about 97% of daily intake) is excreted in the urine, preserving the normal daily equilibrium (28). Thus urinary iodine concentration (UIC) has been considered a sensitive indicator of recent iodine intake (29–31). The status of iodine nutrition can be divided into four stages based on UIC according to WHO iodine recommendations: UIC < 100 μg/L (insufficient), 100–199 μg/L (adequate), 200–299 μg/L (above requirements), and ≥ 300 μg/L (excessive) (32, 33). To adjust for the influences from dilution of the urine, the proportion of urinary I/Cr is also used to evaluate the iodine status (30). Creatinine-adjusted UIC: < 85 μg/g Cr (deficiency), 85–219 μg/g Cr (adequate), and ≥ 220 μg/g Cr (excessive). So as a satisfying bioindicator of the iodine level (34, 35), UIC and urinary I/Cr ratio have been examined in various epidemiological studies aimed to clarify the association between iodine intake and PTC risk.

Therefore, we overview the standpoints from relevant epidemiological studies and experiments to clarify the correlations between iodine nutrition and PTC.

## Epidemiological studies

### Effect of iodine intake on thyroid cancer

Mandatory universal salt iodization (USI) has been put into practice since the 1990s (33), which meets the iodine requirements and gained notable success in preventing iodine deficiency in the general population (36). The ensuing question is whether iodine affects the onset of TC. Numerous epidemiological researches have evaluated the relationship between iodine intake and TC, and have presented a variety of views. Although some studies supported that there is no clear association between iodine nutrition and TC (37–39), a large number of studies these years have provided evidence for the relationship. Most studies were affected by many factors such as ethnic differences, diet customs (23), lifestyle, complications, and other environmental factors which can influence the development of TC. For example, the occurrence rate of TC was increased in two areas of high iodine intake: Iceland (40) and Hawaii (41). However, the natural radiation here is higher than in many other areas, so the radiation here can also drive the development of TC, especially in childhood (42, 43). Therefore, these studies cannot offer persuasive evidence to prove that high iodine intake can be a hazardous factor for TC.

Credible evidence was also presented in some epidemiological studies. A 1992 study evaluated the prevalence of TC in patients with goiter in iodine excess and iodine deficiency areas (44), which proved that individuals with excessive iodine intake had a considerably higher risk of TC than individuals in iodine deficiency regions. What’s more, our epidemiological study in 2006 also investigated thyroid diseases over 5 years in three representative regions with insufficient, normal, and excessive iodine intake (45). No cases of TC were diagnosed in insufficient and normal iodine supplementation areas at baseline; however, 10 subjects were found to have PTC in Huanghua, one region with excessive intake of iodine. Between 1999 and 2004, 13 cases of PTC were found in Huanghua but none were identified in the other two areas. A retrospective analysis of the association between daily iodized salt intake and TC conducted by another research group in Hunan province also showed that consuming more than 5 g of iodized salt daily increased the risk of TC (46). Consistent with the above conclusions, a study examining TC trends in populations from three different geographic areas in Thailand between 1990 and 2009 showed an increase in PTC prevalence and a decrease in FTC prevalence as population iodine deficiency levels declined (47).

At the same time, several studies presented other viewpoints. Some studies supported the protective effect of iodine intake on TC risk (48). For example, an ecological study of epidemiology showed that low consumption of iodized salt with mild iodine deficiency may be responsible for the high prevalence of TC in Daishan Country (49). The results of a meta-analysis also suggested that dietary iodine has a protective effect on TC (50). The drawback, however, is the lack of data on iodine intake. French Polynesia, a mild iodine deficiency area, has one of the highest TC occurrence rates in the world, so iodine was suspected to play a part in this phenomenon. In 2012, a case-control study was conducted among the inhabitants of French Polynesia (51), which showed that in this region, higher consumption of seafood and an iodine-rich diet were associated with a reduced risk of TC. However, a limitation of this study that can not be ignored is that their iodine intake was calculated by the amount and composition of the participants’ daily food intake, using a composition table established in metropolitan France, which may be unsuitable for French Polynesia. What’s more, cooking or other factors also have an uncontrollable influence on the final iodine intake, it would be more accurate by measuring 24-h UIC.

What’s more, previous studies have indicated the prevalence of thyroid diseases may raise with both insufficient and overmuch iodine intake (52, 53). A study in 2016 indicated that compared to patients with benign thyroid nodules, TC patients tended to be distributed in UIC < 300 μg/L and UIC ≥ 2500 μg/L, this suggested that UIC may be involved in predicting TC risk in patients with thyroid nodules (54). The study supported that both low and overmuch iodine intakes can be related to TC in the iodine-replete region, so there may exist a U-shaped relationship between iodine intake and TC. Further research in the future also ought to reveal the mechanism of how iodine works and help to guide iodine intake.

A retrospective study based on patients who underwent thyroidectomy at Peking Union Medical College Hospital (PUMCH) from 1986 to 2018 implied that PTC has become the predominant type in TC surgery after USI, while the proportion of other histological subtypes has remained stable during this period (55). Therefore, we focused on the effect of iodine levels on PTC.

### Effect of iodine intake on papillary thyroid carcinoma

Current studies reflected that iodine intake has a significant impact on PTC though some studies did not agree with this conclusion (56). One study that followed TC prevalence before and after iodine prevention in Argentina shows that PTC patients increased significantly after iodine supplementation, PTC/FTC ratio also increased significantly (57). Therefore, it is speculated that high iodine intake may be associated with a high prevalence of PTC (58, 59).

The prevalence of thyroid diseases in Shenyang has also raised obviously with the iodine intake increased since USI was implemented in China in 1996 (60). The diagnosis of TC and the proportion of PTC raised notably, and the proportion of FTC and UTC reduced while the ratio of MTC was not changed after USI. This study did find a correlation between iodine intake and TC, especially PTC, but there are also advances in detection technology and overdiagnosis, which need to be further verified. UIC differences between patients with PTC and nodular goiter were not statistically significant in another study (61), while in female PTC patients, extremely excessive iodine intake was independently related to the increased tumor size. This study supported that high iodine intake may be associated with the increase of tumor volume rather than its oncogenesis. Contrary to the above conclusions, a study conducted in a multiethnic group, investigated dietary iodine exposure among TC women in the San Francisco Bay area and women in the general population and concluded that an increase in dietary iodine is most likely associated with a reduced risk of PTC in those “low-risk” women (women with no risk factors) (62).

## Iodine intake and combined factors

Nowadays, some studies have indicated that the combined effect of iodine and other factors plays a certain role in the occurrence and development of PTC. For example, Bisphenol A (BPA) is a kind of organic material that is widely applied to manufacturing processes (63). It has been reported that as an effective endocrine disruptor, free BPA can inhibit the expression of thyroid hormone-regulated genes by binding to thyroid hormone receptors (64). One study investigated whether BPA levels and excessive iodine intake were linked to PTC (65). The results indicated that the PTC groups’ UIC and Urinary BPA concentrations (UBC) were higher than those in the control group, which suggested that high levels of UBC and iodine intake may be the predictive factors for PTC. What’s more, BPA and iodine may interact with each other through some common pathways in the process of the occurrence and development of PTC.

A 2020 study tested UIC and thyroid function in patients with PTC, patients with benign thyroid tumors, and healthy individuals (66). The median UIC of the PTC and benign thyroid tumor group was markedly higher than that of healthy control groups. The regression analysis in this study also indicated that thyroglobulin antibody (TgAb) was an independent risk factor for PTC (67). What’s more, the association between TgAb and UIC was noteworthy, indicating that excessive iodine in patients with thyroid tumors may affect TgAb, which may contribute to the development of thyroid damage and subsequent malignancy (such as PTC) (68). Another case-control study in 2021 evaluated the cooperative effect of iodine intake and thyroid function on the risk of developing PTC and papillary thyroid microcarcinoma (PTMC) (69), indicating that excessive iodine intake using creatinine-adjusted UIC and high free T4 levels may have a synergistic effect on PTC and PTMC. Therefore, it is of interest to consider thyroid function in addition to iodine intake to predict the risk of PTC and PTMC. This also suggests that the combined effect of UIC and hormones on PTC risk needs to be verified in future larger studies.

### Iodine intake and lymphatic metastasis in papillary thyroid carcinoma

A study in 2014 assessed the median urine iodine (MUI) of participants in Qingdao (70) and found that patients with benign thyroid nodules (MUI = 331.33 μg/l) and patients with PTC (MUI = 466.23 μg/l) had higher iodine intake than people in the control (MUI = 174.30 μg/l), which was in the iodine-replete region. In terms of MUI level, PTC patients with lymph node metastasis were higher than PTC patients without lymph node metastasis. The clinical data of 359 PTC patients who underwent surgical treatment in PUMCH from May 2015 to November 2020 were retrospectively analyzed (71). Consistent with the conclusions of previous studies, they demonstrated that low iodine was a protective factor for central lymph node metastasis in PTC, which indicated that iodine may not only be a promoter of tumorigenesis, but also a predictive factor for the aggressiveness of PTC. Another study also raised the point that high iodine intake does not seem to be a trigger, but may be a weak promoter for PTC progression in women patients, which needs further validation (72). The above data are consistent with most epidemiological studies that show an association between high iodine intake and PTC and its aggressiveness.

#### Iodine intake and BRAF mutation in papillary thyroid carcinoma

The familiar PTC mutation types include BRAF mutation, RET rearrangement, and RAS mutation. Among these alterations, BRAF mutations occur most frequently in PTC (73–75). Some studies proved that the BRAF V600E mutation plays a part in the biological behaviors of PTMC (≤ 1 cm) and small PTC (1–1.5 cm) (76). However, the correlation between these alterations and iodine intake remains controversial (77). Kowalska’s institution diagnosed an increased prevalence of BRAFV600E alterations in PTC, then they speculated that changes in iodine intake might contribute to the increased prevalence of TC (78). To clarify the above perspective, Guan and her team (79) assessed and compared the prevalence of the T1799A BRAF mutation in 1,032 PTC patients from five areas with different dietary iodine content in China. This study indicated that the frequency of BRAF mutation and the tumorigenesis of PTC are cogently associated with high iodine intake. The BRAF mutation was also confirmed to be a prognostic marker of PTC. Another study in Korea also investigated the correlation between iodine intake and BRAF mutation in PTC patients (80). BRAF mutation was the lowest in the 300–499 μg/L UIC group, which was different from that in the rarely low iodine intake (UIC < 300 μg/L) and excessive iodine intake (UIC ≥ 500 μg/L) groups, confirming that UIC can be used as the predictor of BRAF mutation in PTC. Their results verified the U-shaped curves again.

Some studies hold contrary views. For instance, one 2016 study conducted molecular analyses of two differentiated TCs, PTC, and FTC in two countries with different iodine intake: the iodine-rich country (Japan) and the iodine-poor country (Vietnam) (77). Their study indicated that there was no difference in genetic mutations between patients from iodine-rich and iodine-poor countries, the conclusion may support that iodine status does not influence the genetic changes of PTC and FTC. Another study also investigated the iodine intake of PTC patients with or without BRAFV600E mutation and that of healthy participants in 2018 (81). Though their results indicated that iodine status differs significantly between PTC patients and healthy participants, the correlation between iodine status and BRAF alteration was not statistically significant.

Many epidemiological studies and meta-analyses showed inconsonant conclusions because of dietary information bias, measurement error, and differences in ethnic groups and regions. It is also uncertain whether there is publication bias (82) or other factors influencing thyroid carcinogenesis (83). So definitive epidemiological studies are still warranted in the future.

## *In vitro* studies

Most of the current studies focused on epidemiological investigation, the molecular biological effect of iodine promoting PTC is unclear until now. Here we review the mechanism of iodine-induced biological behavior of PTC cells. Studies have supported the protective function of excessive iodine on thyroid follicular cells through specific pathways. For instance, RET, a proto-oncogene involved in the carcinogenesis of PTC, can be activated by the fusion of the tyrosine kinase domain with the 5′ region of another gene. This process can produce chimeric products, collectively known as RET/PTC (84–86), leading to the activation of the MAPK pathway, which plays a part in driving PTC. So a study once evaluated the effect of high iodine concentrations on RET/PTC3-activated thyroid cells and indicated an antioncogenic role for excess iodine during thyroid oncogenic activation (87). Consistent with this viewpoint, another study in 2014 cultured thyroid follicular cells with doxycycline for 2 days, with or without 10 μM sodium iodide (88), then they found that high iodine inhibited miR-19, the newly discovered regulator of Smad4, which was activated by BRAFV600E, and restored the response to TGF-β signaling *via* the Notch pathway. This study indicated that iodine has a protective influence on thyroid cells, alleviating microRNA deregulation mediated by the BRAF oncogene, which contributes to the understanding of the physiological role of iodine on PTC. In addition, a recent study found that BRAF kinase can induce autophagy in PTC cells to participate in anti-apoptosis, and promote cell proliferation and migration under high iodine concentration (89), which support the view that high concentration of iodine can inhibit cell proliferation and promote cell apoptosis and migration.

Other studies have shown that excess iodine has adverse effects on thyroid cells. A study found that with a high iodine treatment, the miR-422a/MAPK1 pathway was complicated in the procedures of cell migration and proliferation, thus regulating tumorigenesis (90). With a high iodine concentration (100 μM), the MAPK1 signaling pathway was activated significantly in thyroid follicular epithelial cells, which means that in normal thyroid cells, high iodine may lead to the imbalance of the miR-422a/MAPK1 pathway. Considering that they only conducted functional experiments in two iodine concentrations, more studies are in the future.

Several studies have found that iodine has a double influence on thyroid cells’ behaviors, depending on the iodine concentration. A study assessed the influences of different iodine concentrations on the proliferation and migration of two well-differentiated thyroid cell lines *in vitro* (91). The results supported that when iodine concentration was at a certain level, it could play a role in promoting the proliferation of thyroid cells. Iodine under 1.0 × 10^–3^ mM promotes the growth of thyroid cells while iodine higher than this concentration has the opposite effect. Besides, the mRNA level of VEGF-A was up-regulated in thyroid cells cultured in low iodine concentration (1.0 × 10^–5^, 1.0 × 10^–4^, and 1.0 × 10^–3^ mM) and down-regulated in thyroid cells cultured in high iodine concentration (1.0 × 10^–2^ and 1.0 × 10^–1^ mM), which indicated that the Akt, Erk, and the cytokine VEGF-A are the important mechanisms. However, iodine concentration in the human thyroid is usually from 1.0 × 10^–6^ to 1.0 × 10^–5^ mM, so in the human body, the high level of iodine intake may promote the proliferation and migration of PTC cells. Another study in 2019 also illustrated this dual effect, they investigated how iodine affected the physiological features of TC cells *in vitro*, including proliferation and apoptosis (92). Compared with the control group, extra-high doses of iodine (1.0 × 10^–3^ mol/l) inhibited cell proliferation and promoted cell apoptosis, while extra-low doses of iodine (1.0 × 10^–4^–1.0 × 10^–8^ mol/l) showed opposing effects. Their results also indicated that the level of SPANXA1 was increased in cells treated with a certain concentration of iodine. The SPANXA1 (93) can also be one of the key genes, which enhanced the process of tumor growth in cells treated with an extra-low dose of iodine. Cell proliferation can be promoted by high expression of SPANXA1 while cell apoptosis can be inhibited by SPANXA1. In addition, PI3K/AKT was supposed to be a key signaling pathway through which SPANXA1 mediates its effects. Thus, SPANXA1 can be a biomarker in PTC and help in guiding dietary plans for patients with TC, which remind us that patients’ iodine intake should be restricted.

Though these studies suggested some possible mechanisms for how iodine affected thyroid carcinogenesis, many other confounding factors cannot be ruled out. The effects of iodine on PTC patients are also complex and influenced by many chemical agents *in vivo*, so it is hard to clarify the interaction and feedback mechanisms of so many hormones by conducting cell experiments. Therefore, more *in vivo* studies are needed to clarify the function and mechanism of iodine on PTC.

## *In vivo* studies

Animal studies examining the effect of different levels of iodine intake on the development of PTC were still rare. But earlier studies have shown that the development of iodine deficiency can cause PTC. The long-term effects on the thyroid with low iodine intake were assessed in 98 Sherman albino female rats (94), iodine deficiency was shown to be attributed to the production of tumors in thyroid glands. One study also found that iodine deficiency can cause goiter, hyperplasia, or malignant change as iodine deficiency time goes on (95), which also speculated that iodine deficiency can lead to reduced thyroid hormone synthesis, while the increased TSH drove chronic overstimulation of the thyroid. Proliferating thyroid cells, meanwhile, can also be more susceptible to radiation, chemical carcinogens, and oxidative stress, so more genetic mutations will show up in these cells. In addition, thyroid hyperplasia caused by insufficient iodine can lead to the change of chromosomes in the thyroid and increase the number of aneuploid cells in rats (96). Therefore, it is speculated that chronic stimulation in iodine deficiency may be one of the vital mechanisms of PTC. However, another study proved the U-shaped relationship by investigating the influence of iodine intake on p14ARF and p16INK4a expression of PTC in rats (97). This study suggested that both low and high iodine intake can decrease the expression of p14ARF and p16INK4a and drive tumor development.

The association between human iodine intake and PTC still cannot be explained because iodine deficiency or excess is much more severe in most animal models than in the human diet.

## Discussion

Over these years, the occurrence rate of TC, especially PTC is increasing significantly in the world (1). Although overdiagnosis has been reported to increase the prevalence of PTC (98), there has also been a true increase. It is therefore meaningful to illustrate the role of these suspected risk factors, especially iodine intake. TC has been reported in iodine deficiency areas in earlier years. The prevalence of PTC also increased after iodine intake increased due to salt iodization (99) and iodine supplementation. In contrast, iodine was a protective factor for PTC in some studies, which lead to controversy about the correlation between iodine intake and PTC. Previous epidemiologic studies’ results could be influenced by different test standards, study methods, dietary habits, measurement errors, information bias or so many other factors. For example, it is difficult to measure 24-h UIC, the gold standard for iodine intake (31). Therefore, some studies may use random spot urine UIC as an alternative indicator. While the studies *in vitro* or *in vivo* cannot reflect the true human iodine status, the evidence is far from sufficient. Hence it is still unclear the true iodine interval that directly induces PTC development, or indirectly contributes to PTC risk through interaction with other factors.

Many previous studies have addressed this controversial issue, showing that different iodine nutritional status has different effects on the development of PTC. We discuss their views in this article and summarize the basic information in Table 1 to help readers think objectively. Taking the present studies into consideration, we speculate that the relationship between iodine nutrition and PTC may be intricate and the effect of iodine should be considered dose-based. Besides, the combined action of more variables are ought to be considered in the research of iodine and PTC.

**TABLE 1:** Characteristics of the studies included in this review.

	First author	Publication year	Study period (year/month)	Location	Sample size (*n*)	Research type
Prevalence	Belfior et al. (44)	1992	1980–1990	Italy	5637	Retrospective study
	Teng et al. (45)	2006	1999–2004	China	3018	Prospective study
	Wang et al. (46)	2021	2017/01-2019/12	China	51637	Retrospective study
	Mitro et al. (47)	2016	2001–2009	Thailand	2749	Prospective study
	Zhang et al. (49)	2019	2014–2018	China	2495	Prospective study
	Clero et al. (51)	2012	1979–2004	France	600	Retrospective study
	Kim et al. (54)	2016	2010/11-2013/05	Korea	1170	Retrospective study
	Zeng et al. (55)	2020	1986–2018	China	34213	Retrospective study
	Dong et al. (60)	2013	1992/01-2009/12	China	1239	Prospective study
	Zhao et al. (61)	2017	2013/11-2015/03	China	2041	Retrospective study
	Horn-Ross et al. (62)	2001	1992–1998	America	1166	Retrospective study
	Zhou et al. (65)	2017	2013/02-2013/09	China	261	Retrospective study
	Hou et al. (66)	2020	2017/01-2019/03	China	506	Retrospective study
	Kim et al. (69)	2021	2010/04-2014/12	Korea	946	Retrospective study
Lymphatic metastasis	Wang et al. (70)	2014	2010/06-2011/06	China	460	Retrospective study
	Zeng et al. (71)	2021	2015/05-2020/11	China	359	Retrospective study
	Zhao et al. (72)	2019	2013/11-2018/02	China	4040	Retrospective study
BRAF mutation	Vuong et al. (77)	2016	2006–2014	Japan	194	Retrospective study
	Kowalska et al. (78)	2016	2000–2013	Poland	723	Retrospective study
	Guan et al. (79)	2009	–	China	1032	Cross-sectional study
	Kim et al. (80)	2017	2010/11-2015/03	Korea	215	Retrospective study
	Lee et al. (81)	2017	2015/03-2015/12	Korea	300	Retrospective study

The relationship between iodine and PTC is complex, we are still unclear about the specific role of iodine, let alone the mechanism of its activities due to the disagreement of current research results. The present studies have yielded mixed results which indicated that iodine intake may influence the development or progression of PTC, change the proportion of several subtypes of TC in the crowd, or affect the invasiveness of PTC especially lymphatic metastasis and BRAF mutation. These data provide further evidence supporting that it makes sense to achieve the appropriate level of iodine intake to satisfy the body’s normal nutritional needs while avoiding either deficient or excessive iodine supplementation.

## Author contributions

WT supervised the work. XZ drafted the manuscript. FZ provided the major technical support. QL and CF assisted in the literature review. All authors contributed to the article and approved the submitted version.
